# Big Data Warehouse for Healthcare-Sensitive Data Applications

**DOI:** 10.3390/s21072353

**Published:** 2021-03-28

**Authors:** Arsalan Shahid, Thien-An Ngoc Nguyen, M-Tahar Kechadi

**Affiliations:** School of Computer Science, University College Dublin, Belfield, Dublin 4, Ireland; an-thien-ngoc.nguyen@ucdconnect.ie (T.-A.N.N.); tahar.kechadi@ucd.ie (M.-T.K.)

**Keywords:** big data representation, healthcare data, big data security, privacy-aware models

## Abstract

Obesity is a major public health problem worldwide, and the prevalence of childhood obesity is of particular concern. Effective interventions for preventing and treating childhood obesity aim to change behaviour and exposure at the individual, community, and societal levels. However, monitoring and evaluating such changes is very challenging. The EU Horizon 2020 project “Big Data against Childhood Obesity (BigO)” aims at gathering large-scale data from a large number of children using different sensor technologies to create comprehensive obesity prevalence models for data-driven predictions about specific policies on a community. It further provides real-time monitoring of the population responses, supported by meaningful real-time data analysis and visualisations. Since BigO involves monitoring and storing of personal data related to the behaviours of a potentially vulnerable population, the data representation, security, and access control are crucial. In this paper, we briefly present the BigO system architecture and focus on the necessary components of the system that deals with data access control, storage, anonymisation, and the corresponding interfaces with the rest of the system. We propose a three-layered data warehouse architecture: The back-end layer consists of a database management system for data collection, de-identification, and anonymisation of the original datasets. The role-based permissions and secured views are implemented in the access control layer. Lastly, the controller layer regulates the data access protocols for any data access and data analysis. We further present the data representation methods and the storage models considering the privacy and security mechanisms. The data privacy and security plans are devised based on the types of collected personal, the types of users, data storage, data transmission, and data analysis. We discuss in detail the challenges of privacy protection in this large distributed data-driven application and implement novel privacy-aware data analysis protocols to ensure that the proposed models guarantee the privacy and security of datasets. Finally, we present the BigO system architecture and its implementation that integrates privacy-aware protocols.

## 1. Introduction

With a rise in income prevalence rates worldwide, almost 7.8% of boys and 5.6% of girls suffered from childhood obesity in 2016 [1]. Since obesity is a major global public health problem [2], with significant cost implications both to the individual and society at large, strong measures must be taken to intervene in childhood obesity. Children with obesity are prone to experience a range of health issues that are both physical and psycho-social [3]. Obesity also contributes to critical health problems such as Type 2 Diabetes and Coronary Heart Disease that develop in childhood [4].

During the last two decades, obesity aetiology has been thoroughly studied in the biomedical sciences, i.e., terms of genetic variation. However, genetic variation among people has been discovered to elucidate nearly 1.5% of inter-individual variation in body mass index (BMI) [5]. Existing epidemiological methods have largely explored the obesity problem as a risk factor for non-communicable disease but have not studied it as an outcome of behaviour within an obesogenic environment [6]. With a lack of reliability of pharmacological interventions and the invasiveness of surgical procedures, an increasing reliance is being put on behavioural remedies.

Interventions targeting various aspects of children’s behavioural patterns, such as, how they eat, how they move, how they interact within their environment, and how they sleep [7] can have a positive outcome towards obesity within children [8,9]. Unfortunately, most global public health actions are limited to indiscriminate ‘blanket policies’ and the profound lack of common monitoring and evaluation framework does not help either [10].

During the last decade, the advances in the area of mobile and wearable devices gave rise to a new generation of innovative healthcare research [11]. Innovative endeavours such as SPLENDID EU project [12] push the boundaries in the use of technology in everyday life to screen monitor and normalise behaviours related to the progression of obesity. In parallel, new achievements in the field of Big Data collection, processing, and analysis allow the expansion of such efforts on a larger scale, allowing the citizens to be actively engaged in reshaping health policies at regional levels. Big data collection aims to explore causal relationships between behaviour, the built environment, and obesity.

Recent developments in the fields of behaviour change science, public health, clinical paediatrics, ICT, citizen science and Big Data analytics can be harnessed to implement multidisciplinary research projects to address the prevention and treatment of child and adolescent obesity at a population level. The H2020 project “BigO: Big Data Against Childhood Obesity” (http://bigoprogram.eu, accessed on 21 March 2021) is one example of such research efforts. BigO project aims to redefine the way strategies that target childhood obesity prevalence are deployed in European societies. These new strategies use citizen-scientist data collection methods that gather large-scale data using different mobile technologies (smartphone, wristband, mandometer) [13]. The main goal is to create comprehensive predictive models of the obesity prevalence dependence matrix to study the effectiveness of specific policies on a community and the real-time monitoring of the population response. Data is uploaded by citizen scientists onto the BigO cloud infrastructure for aggregation, analysis, and visualisation. The large-scale data acquisition in BigO allows researchers to create models for analysing behavioural risk factors and predicting obesity prevalence, through associations with community behavioural patterns and local obesity prevalence.

Since, BigO involves monitoring and storing of personal data (photographs of meals, beverages, food and drink advertisements, and GPS data) related to the behaviours of a potentially vulnerable population (i.e., children and adolescents), the data representation, security, and access control is challenging. In this study, we first developed the necessary components of the BigO system dealing with data access and storage, including the definition and implementation of their interfaces with other system components. This has significantly facilitated data aggregation, data analysis, and visualisation, while adhering to data privacy of individuals and security of the whole system. More precisely, we developed a three-layered data warehouse architecture for BigO. which includes a back-end layer, an access control layer, and a controller layer. The back-end layer consists of a database management system for data collection, de-identification, and anonymisation. The role-based permissions and secured views are implemented in the access control layer. Finally, the controller layer regulates the data access protocols.

The sharing of sensitive personal data in the healthcare sector is regulated by rules and laws such as Directive 95/46/EC of the European Union and HIPAA of the United States [14,15]. Two commonly acceptable approaches for privacy protection: (a) taking patient consents, and (b) anonymisation of personal data. The first approach maintains the correctness of the analysis results but is time-consuming, inflexible, and more prone to data breaches when employed to collect data from a large set of patients [16]. The anonymisation approach, however, is more flexible but impacts the data quality and analysis; especially in the case of high-dimensional data [17].

Considering the high complexity of the BigO data, we first introduce the data representation schema and the storage models taking into account the privacy and security. The data privacy and security plans are devised based on the types of collected personal data in the data storage, data transmission, and data access protocols. We discuss in detail the challenges of privacy protection and implement efficient privacy-aware data analysis protocols to the privacy for children. Note that the system is designed to recognise and disregard redundant individual information. Finally, we implemented the BigO system architecture that integrates privacy-aware protocols.

The rest of the paper is organised as follows: Section 2 presents the data anonymisation challenges and system requirements for healthcare data analysis along with existing privacy-aware architectures. This is followed by an overview of the BigO system and the data collection methods with the data flow based on the type of users in Section 3 and Section 4, respectively. In Section 5, we present the BigO data warehouse architecture and the big data representation in the storage system. Section 6 discusses the security and privacy considerations for BigO along with a privacy-aware data analysis protocol. Section 7 is devoted to the system implementation and how the privacy-aware protocol is integrated in the system architecture. In Section 8, we provide discussions on the pros and cons of our work and highlight the future research directions. Finally, Section 9 concludes the paper.

## 2. Healthcare Data Privacy

We first present a brief overview of data anonymisation challenges in data-driven healthcare systems and then we list the necessary privacy and security requirements for personal data monitoring, sharing, and analysis in healthcare systems. We further present a brief review of existing systems and evaluate them based on these requirements.

### 2.1. Data Anonymisation and Sharing

The data attributes (or dimensions) in a typical healthcare dataset can be divided into four categories based on their sensitivity and their relation with the subject; (1) sensitive, (2) non-sensitive, (3) identifiable, and (4) quasi-identifiable. Table 1 shows that, alongside clearly sensitive and identifiable attributes, there are a number of features called quasi-identifiers that can be combined to identify specific individuals. Preserving the privacy of a piece of data that is sensitive, identifiable, or quasi-identifiable is a critical challenge. Data anonymisation maintains a low confidence threshold for linking the sensitive information to an individual [18].

As a consequence of data sharing Record linkage [18] and attribute linkage [19] are two main types of attacks, which are commonly used. Both of these can be prevented using the privacy models, such as *k*-anonymity [20] and *ℓ*-diversity [19]. Some of the extended versions of these privacy models, including (X,Y)−anonymity,LKC−privacy, and t−closeness [18]; can be implemented using various anonymisation operations, such as noise addition, generalisation, shuffling, and perturbation [21]. However, some of these operations, including perturbation and cell generalisation, affect the quality of datasets and their usability for knowledge discovery (by means of mining algorithms) or information retrieval.

### 2.2. Healthcare Data Requirements

The following are the necessary requirements of healthcare datasets, for which, on one hand, should be protected and kept private and on the other should be able to be analysed to extract useful knowledge that can advance healthcare research and practices. The key idea is to create an environment where the private and sensitive data be analysed without revealing the identity of individuals.

- Privacy: This is essential mainly in the healthcare sector. Patients’ records and their data attributes are very exposed to attacks. So it is imperative to put in place protection mechanisms to preserve the privacy of patients and individuals when sharing healthcare datasets.
- Data Quality: High-quality data is essential for data mining and analysis (as no quality data leads to no quality results). Therefore, shared data should maintain good attribute values that are detailed enough to serve the purpose of the mining and analysis. One must also carefully take into account the curse of high-dimensionality [17] and maintain the truthfulness at an individual record level.
- Flexibility: Privacy protection should be flexible enough for various analysis tasks and mining techniques. The ideal approach is to implement privacy-preservation solutions independently from the mining algorithms and research purposes.
- Compatibility: Privacy-preserving models should comply with and support the system reference architecture.
- Utility: Provide a level of support to allow researchers to re-visit patient data following appropriate access control and ethics mechanisms.

### 2.3. Existing Data Privacy-Enhancing Techniques

**Figure 1:**
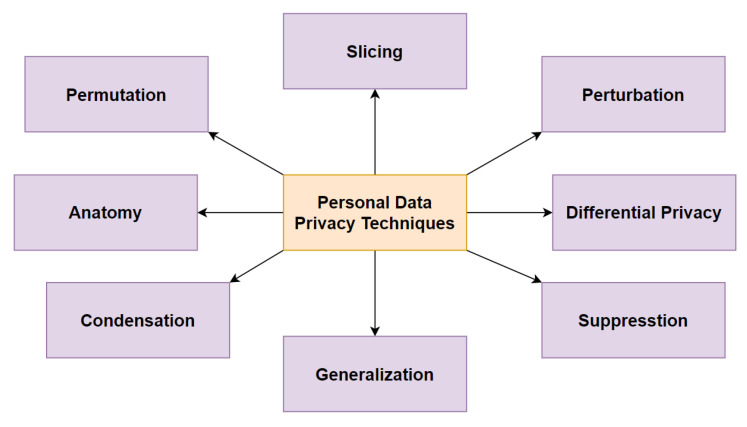
A taxonomy of personal data-privacy techniques. Adopted from [22].

Data privacy is a complex and multidimensional concept that has become a critical threat in the modern-day age. It is defined in a legal, philosophical as well as in a technical context. Personal information privacy techniques aim to address the problems regarding the private information of a person and its exposure. Figure 1 provides a taxonomy view of existing data-privacy techniques dealing with data anonymization and randomization to ensure the privacy of sensitive information.

The existing healthcare systems (Section 2.4) lack the integration of privacy-aware protocols in three main dimensions including storage, transmission, and processing. Some theoretical frameworks have introduced data anonymisation methods for maintaining the individuals’ privacy, but they degrade the quality of data analysis tasks.

In the following, we review and evaluate existing frameworks based on the aforementioned requirements.

### 2.4. Comparative Analysis of the Existing State-of-the-Art Healthcare Systems

We first review the recent European research projects tackling obesity prevention. Project ToyBox [23] presented and holistic, cost-effective, and family-involved schemes to support obesity prevention in early childhood. Project MOODFOOD [24] explored the prevention of depression by looking at the patients diet, food eating behaviour, and obesity. The project studied over 990 participants from the Netherlands, United Kingdom, Germany, and Spain. The project performed correlation studies with patients’ depression history and eating bahaviour. The results suggested that unhealthy eating activities are the leading factors for depression. The EU project SPOTLIGHT [25] conducted a systematic study to identify individual-level obesity intervention factors. The project reviewed the role of social and environmental factors related to obesity and conducted the qualitative analysis of multi-level interventions considering the reach, effectiveness, implementation, and adoption. Project Daphne [26] developed a data-as-a-service healthcare platform for tackling health, weight, physical activity, and lifestyle by linking the technology platforms with clinical supports. It should be noted that none of the aforementioned projects, which dealt with obesity prevention, provided privacy and security-aware protocols to handle sensitive personal information of the patients.

The collection of personal data using sensor technologies is valuable to understand and cure various healthcare problems such as obesity. However, with the rapid increase in sensor technologies, sensitive personal data such as users’ activities and characteristics suffers from privacy threats. A set of rules such as the General Data Protection Regulation (GDPR) [27] act as a guide on how companies should collect and share personal data of EU citizens by putting the privacy-aware solutions in-place. To comply with the GDPR protocols, it is essential to obtain user consent, especially in the healthcare sector. Rantos et al. [28] proposed ADvoCATE to help users control the consents related to access their personal data collected from sensors and wearable technologies. The proposed solution also guides the data controllers and processors to meet the GDPR requirements. Larrucea et al. [29] presented the GDPR compliant architecture reference models and consent management tools for the healthcare industry. The authors identified potential security and privacy threats in the architecture models and used data hiding tools to ensure privacy while sharing health records. Mustafa et al. [30] presented a comprehensive review of privacy requirements for mobile health applications in the context of GDPR and evaluated the privacy requirements of the system that supports monitoring, early diagnoses, and detection of anomalous situations among patients suffering from Chronic Obstructive Pulmonary Disease (COPD).

There are a few theoretical studies that have also been conducted, which propose to develop data warehouse architectures in healthcare settings. Sahama et al. [31] proposed a data warehouse architecture that tackles the data integration issues. The authors highlighted the need for exploring secure access to data warehouse models while respecting the healthcare decision support systems by using evidence-based, case-based, and role-based data structures. Ali Fahem Neamah [32] proposed a flexible and scalable data warehouse for building an electronic health records architecture. The authors highlighted some issues of the system to support mobile application development, including compatibility with very large platforms and devices. Poenaru et al. [33] proposed advanced solutions in the form of data warehousing for medical information storage to tackle some issues, such as complex-data modelling features, classification structures, and data integration. All of the aforementioned data warehousing solutions do not take care of any privacy and security issues for data storage, access, and analysis.

Along with improving the data collection and integration protocols in healthcare systems by taking the GDPR complaint patients consents, it is equally essential to store personal data in a secure and privacy-aware manner without disturbing the quality of the data for carrying out periodic analysis tasks. Therefore, data anonymisation protocols have been implemented in a number of healthcare frameworks. However, within the big data domain; having several attributes, which may also be considered as quasi-identifiers, data anonymisation becomes non-trivial and results in a large amount of information loss [17]. Datafly, proposed by Sweeney et al. [34], uses the data receivers’ profiles and global data requirements to perform data anonymisation. However, the integration of Datafly in healthcare systems is difficult due to its implementation as a standalone program. Furthermore, it does not take into account the attribute linkage attacks and the curse of high-dimensionality. The extensions to Datafly, as proposed in [35] tackle the privacy problems but the compatibility and data quality remain the same. Agarwal et al. [36] proposed Hippocratic by providing the data disclosure management protocols and services. The framework supports access control, anonymisation, and auditing but lacks flexibility, privacy-awareness, data quality, and compatibility. When evaluated for various analysis tasks, Hippocratic loses the truthfulness at the record level and causes wrong mining results [18] because of the anonymisation operations, such as perturbation and cell generalisation. Prasser et al. [37] proposed an ARX framework that addresses the privacy issues by implementing privacy-aware models but lacks the data quality by not considering the impact of high-dimensionality.

Nguyen et al. [38] presented privacy-aware protocols for Electronic Health Record (EHR) systems that use secured views along with a high-level middleware architecture. Tran et al. [39] proposed a model-driven distributed architecture for security in healthcare data storage and analysis that controls the access permissions to sensitive data and transmission control between distributed nodes. These two strategies are efficient and need further investigation.

### 2.5. Summary

Recent research studies in the healthcare sector have focused on developing GDPR compliant protocols in the form of a consent management system for data collection. Existing state-of-the-art healthcare data warehousing systems tackle various issues, such as flexibility, scalability, data integration, and software system compatibility. However, these systems lack the integration of privacy and security-aware protocols for data storage, access, and analysis. A number of frameworks have introduced data anonymisation methods for maintaining the individuals’ privacy, but when evaluated for the analysis tasks yields incorrect mining results. The quality of data is further degraded when the impact of high-dimensionality is not taken into account. Some works presented the privacy-aware protocols by implementing the secured-views and access controls for storing EHRs, but they require further investigations in real-world healthcare settings.

To summarize, healthcare data warehouse architecture should implement privacy-aware protocols for monitoring and storing of personal data. The main objectives while constructing a healthy healthcare dataset include data protection and privacy, and suitability to analyse and mine insightful knowledge to improve healthcare research and practices. In other words, data-driven healthcare platforms should strive to develop an environment where private personal and sensitive data could be analysed without revealing the individuals’ identity.

The BigO project aims to collect large-scale data of over 25,000 individuals in schools and clinics for developing effective obesity prevention policies. Such large-scale personal data collection, storage, and processing require research and development towards implementing strong privacy and security protocols and frameworks within the data warehouse architectures. In this work, we propose a three-layered data warehouse architecture which includes:

1. A back-end layer with database management system for data collection, de-identification, and anonymisation of the original datasets.
2. The role-based permissions and secured views are implemented in the access control layer.
3. The controller layer regulates the data access protocols for any data access and data analysis.

We further present the data representation methods and the storage models taking into account the privacy and security mechanisms. The data privacy and security plans are devised based on the types of collected personal, the types of users, data storage, data transmission, and data analysis. Finally, we present the BigO system architecture and its implementation that integrates privacy-aware protocols.

## 3. BigO System—Overview

This section presents the BigO objectives, the data sources and measurements, and the involved stakeholders for understanding the causes of obesity in children.

**Figure 2:**
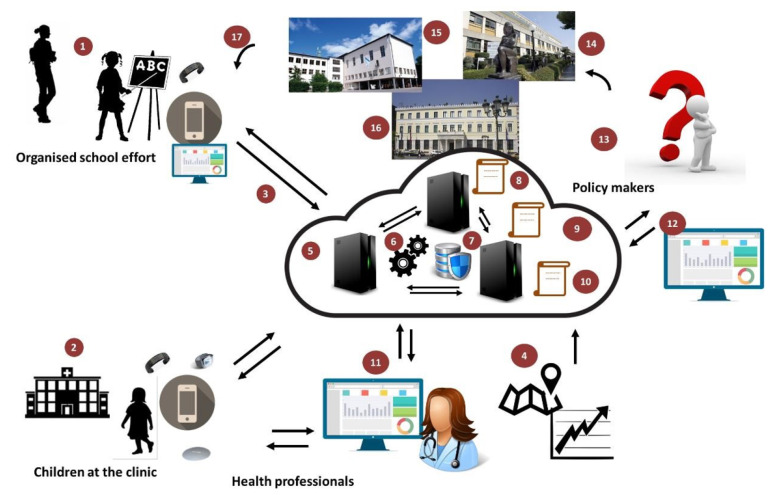
Overview of the BigO system. (1) Citizen scientists. (2) Children are monitored at clinics, through smartwatch and mobile apps. (3) Anonymised and encrypted data transmission. (4) External data sources (maps, POIs, area statistics). (5) BigO cloud data aggregation and processing. (6) Data analytics and visualisation libraries and tools. (7) Secure distributed database storage. (8) Policy advisor service. (9) Policy planner service. (10) School and Clinical advisor services. (11) A clinician using web tools to monitor and guide children. (12) Web tool for policymaker decision support. (13) Policymakers identify childhood obesity conditions. (14, 15, 16) Policies are applied to hospitals, schools, and the community or regional level. (17) Applied policies affect Citizen Scientists, closing the loop and initiating another round of data collection and analysis.

BigO strives to provide an innovative system, allowing the Public Health Authorities (PHAs) to evaluate their communities based on their obesity prevalence risk and to take local actions, based on objective evidence. Figure 2 provides an overview of the BigO platform. Children or adolescents in schools and clinics participate as citizen scientists, to provide data through sensors of their mobile device (phone or smartwatch). They interact with the system through mobile phone and/or smartwatch applications, the school portal (via their teachers), the clinical portal (via their clinicians), and the online community portals. The online web portals provide insights from the data gathered by the BigO platform. They also allow the students to visualise simple summaries of the data and quantify their contribution to the BigO initiative, and how to act against obesity. The mobile apps and web portals act both as data collectors as well as engagement mechanisms, for helping users to contribute with their data and understand why and how their data is useful in this case. Their data is used for measuring the behavioural indicators and Local Extrinsic Conditions (LECs) of the environment, which are relevant to childhood obesity. To measure LECs, the data is also collected from publicly available external sources, such as maps, Geographic Information System (GIS), and statistical authority services. The collected information is processed using the BigO analytics, visualisation, and simulation engines, which extract meaningful indicators describing behaviours, the environment, as well as models of their relations. The resulting measurements support the operation of the Policy advisor, Policy Planner, and School and clinical advisor services of the system.

To summarise, from a technical perspective, BigO measures and study two main factors: (1) local extrinsic conditions and (2) personal behavioural patterns. Data gathered from these factors is interpreted by the BigO users listed below:

1. Children and adolescents within an age band (9–18 years old) who are data providers as:
  - School students, via organised school efforts on projects around physical activity, eating, and sleep.
  - Patients attending obesity clinics.
  - Individual volunteers.
2. Teachers running the organised school efforts with students.
3. Clinicians treating patients in clinics.
4. Public Health officers (researchers or policymakers) evaluating the children/adolescents behaviour indicators in a geographical region in the combination of Local Extrinsic Conditions (LECs) relevant to obesity.
5. Administrators for school, clinic, and the whole BigO platform.

**Figure 3:**
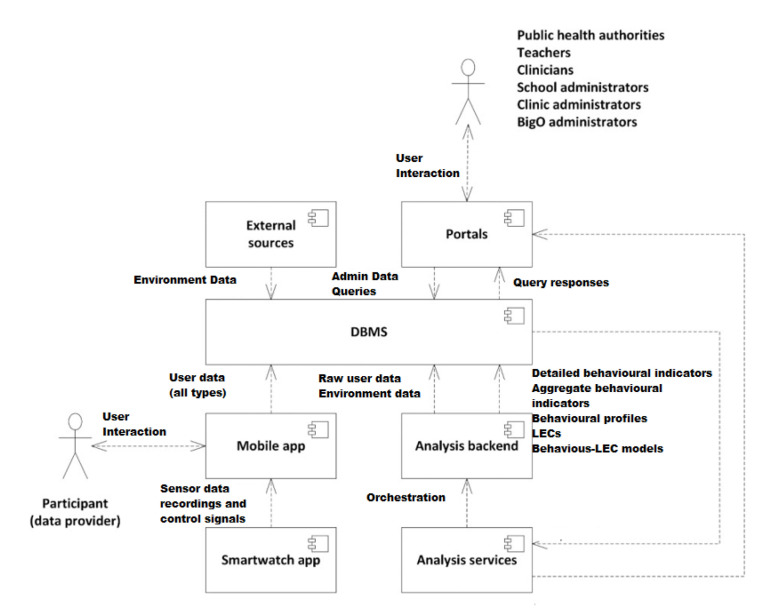
BigO information flow.

Figure 3 summarises the data flow based on the aforementioned user groups. Initially, teachers and clinicians insert data into the systems (initial users registration). The children, both actively (by recording food ads or meals) and passively (by automatic movement detection) share their data by sending it into the system. All this data is organised and represented in a Database Management System (DBMS) and uploaded into the servers, where it can be immediately viewed, processed, and analysed in the portals.

## 4. BigO Data Collection

**Figure 4:**
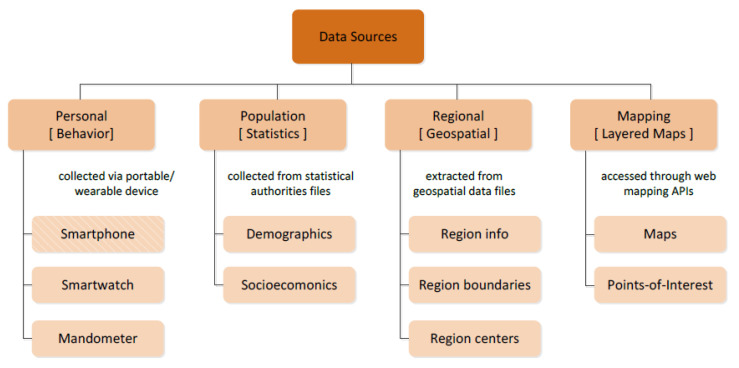
Hierarchical classification of BigO’s required raw data sources. The smartphone is differentiated visually from its siblings, as it is a hybrid raw data source (due to the food advertisement photos).

Figure 4 shows the four main categories of the data that is collected by BigO; (1) personal or behavioural data sources, (2) Population data sources, (3) Regional data sources, and (4) Mapping data sources. These are briefly discussed in the following.

1. Personal data sources (Behavioural): This raw data are collected at the individual level from the citizen-scientists concerning the behavioural patterns that are relevant to the BigO study (e.g., how one moves, eats, sleeps). The raw data in this category are collected from personal portable and/or wearable devices. These sources are further categorised according to the mobile sensory data acquisition device; (i.e., (a) Smartphone, (b) Smartwatch, and (c) Mandometer).
  We combine the devices in three settings based on the requirements of the BigO system that entail data collection and the availability of the peripheral sensors (Table 2).
2. Population data sources (Statistics): The raw data sources contain information characterising the population residing in a given area (countryside, cities, etc.). The data providers include national statistical authorities of the countries involved in BigO. Depending on the type of population statistics, the raw data sources are further classified as (a) Demographic data sources and (b) Socioeconomic raw data sources concerning the population of a city of interest and their administrative regions.
3. Regional data sources (Geospatial): These incorporate geospatial data that are linked with the BigO areas of interest (countries, cities, or administrative city regions).
4. Mapping data sources (Layered Maps): The web mapping data are provided by 3rd party APIs. Depending on the data type, these sources are further classified as a) Maps (i.e., interactive terrain maps) and (b) Points-of-Interest (PoIs).

## 5. Big Data Warehouse Architecture

**Figure 5:**
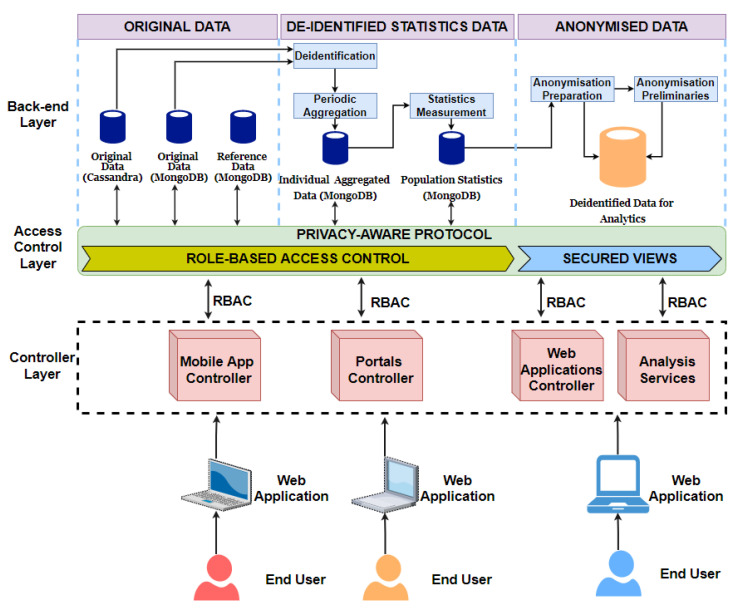
BigO Data Warehouse Architecture.

This section presents the big data warehouse architecture and the data representation methods implemented in the BigO taking into account the data security and privacy. The BigO data warehouse (DW) architecture is shown in Figure 5. The DW consists of various storage systems depending on the sensitivity and the types of accesses required by the data at any stage of its life cycle within the BigO system. It is designed following a three-tier architecture. The three tiers (or layers in this case) are the back-end layer, access control layer, and controller layer.

- Original data: In the BigO system, the raw data is stored following two main schemas. These schemas were implemented using two modern databases; MongoDB and Cassandra. We created two schemas for two different reasons: the first is for security and privacy reason. Separation reduces the risk of data access violation and intentional manipulation. The second is that the two schemas are not used in the same way during the analysis and from various roles. For instance, Cassandra is used for storing time-series data, while MongoDB is used for the rest. Moreover, external sources of data were also used during the analysis, these include authority databases, and national statistical databases.
- De-identified statistics data: All information about users’ identities is removed. This consists of individual aggregated data and population statistics, i.e., the statistical data derived and computed from the original and reference data.
- Anonymised data: In addition to de-identification, we further anonymise the data so that the original data cannot be recovered from the knowledge extracted during the data analysis, using data mining algorithms.

### 5.1. Access Control Layer

This layer includes role-based access control and secured views. The role-based access control checks permissions of the controller as they request accessing the original data and de-identified statistics data based on their roles. The role-based access control only receives the data requests from the mobile app controller or portal controller. Whereas, the secured views control the knowledge extracted from the anonymised data. The secured views receive only data requests from web applications controller and analysis services.

### 5.2. Controller Layer

There are four types of controllers, that is, mobile app controller, portal controller, web applications controller, and analysis services. This layer receives data requests from the users or other components of the system. They check users’ permissions, using tokens and roles of components, whenever they receive the data requests from the users.

### 5.3. Data Storage and Integration

**Figure 6:**
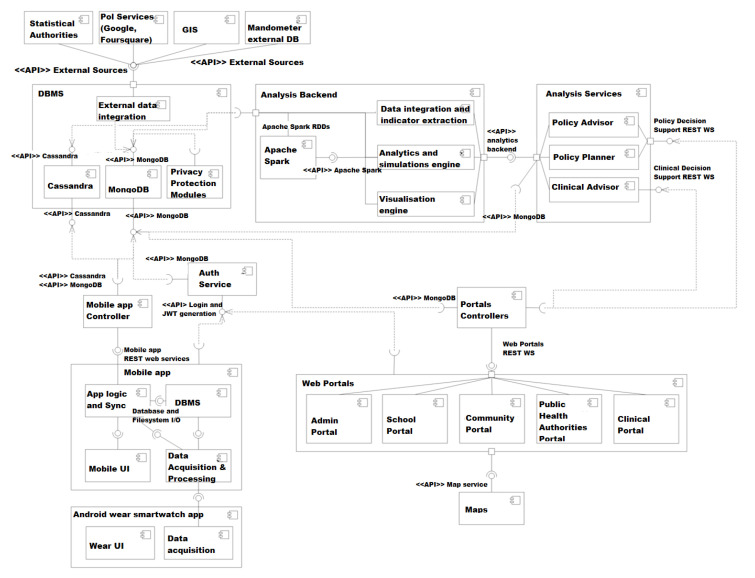
Component diagram of the BigO system.

The storage system that is defined to implement the BigO data warehouse uses MongoDB and Cassandra. MongoDB is the main application database, while Cassandra is used for the storage of large volumes of time series data. The Mobile, Web portal, and Web application controllers directly access the storage system in writing only through REST APIs. These APIs, moreover, implement data integration and consolidation requirements so that the data, collected from different sources, is stored in a unified representation in the data warehouse. Figure 6 shows the flow of data between various system modules.

In addition to the core databases, BigO provides access to a distributed file system that is HDFS of Hadoop. Its purpose is to hold large dataset files that can be directly extracted from the databases, in a convenient format, which can be used by the data analysis algorithms (module). A periodic process daily updates the file contents from the database at a regular time interval. Appendix A.1 and Appendix A.2 present a detailed schema from the core databases. In the following, we discuss the data stored in each one of the core databases.

#### 5.3.1. MongoDB Data

The data stored in MongoDB is collected from four different sources, which are described below:

- Portal controllers: There are five portals in the BigO system; admin portal, school portal, community portal, public health authorities portal, and clinical portal. The data is collected from all these portals, and integrated before storing it MongoDB database.
- Mobile controller: This controller deals with the data collected from either mobile phones or smartwatches. The data is pre-processed, integrated, and then transferred to MongoDB. This data is of particular importance, as it is sensitive and should be handled with care.
- Analysis service: The data analysis component processes the whole Datasets which is stored in two DBs. The analysis component uses a Spark computing environment. The results of the analysis are stored back at the MongoDB database.
- Back-end analysis service: This service accesses the data stored in both DBs to extract the behavioural indicators. These new data attributes are then stored in MongoDB. The back-end analysis service is executed in the Spark environment.

#### 5.3.2. Cassandra Data

This DB mainly stores raw data collected from external sources and mobile apps.

- External sources: The external data sources include individual behavioural data and extrinsic population data. These datasets are directly transmitted to the database through the external data integration modules (Figure 6). For example, data about individual’s devices.
- Mobile app controller: Data from mobile applications collected through smartphone or smartwatch is stored using a mobile app content provider in the DB of the mobile app. The stored data is then synchronised with Cassandra DB. (Figure 6).

## 6. Data Security and Privacy

Privacy and security are fundamental requirements for any information system. Although data security and data privacy are related, they deal with different issues and need different countermeasures. Data security aims to preserve confidentiality, integrity, availability, and non-repudiation. While data privacy prevents shared data from disclosing sensitive information about their corresponding owners. The following data security methods were implemented in BigO.

- Secure storage: The BigO system architecture is implemented using mainstream platforms, such as Cassandra, MongoDB, SQLite database management systems, HDFS file systems of Hadoop, Android, and iOS. These standard systems employ built-in encryption technologies to secure both the system components and the data that they contain. Section 6.1 provide more details about each type of storage.
- Secure communications: The data must be secured when it is transferred between various modules of the system. Communications between BigO modules use secure protocols, such as SSL, TLS, or HTTPS (See Section 6.1 for more details).
- Data access control: Access control is a complex issue in large systems, such as BigO. Therefore, we implemented a full solution for access control consisting of consistent policies, clear and powerful mechanisms for registration, authentication, and authorization. They are summarised in the following:
  - Mobile app storage: In BigO, the mobile app data storage can be accessed only by the back-end of that mobile phone and contains only personal data of its owner. Therefore, no fine-grained access control is required in this context. It uses a simple access control based on username and password.
  - Auxiliary file storage: This is a temporary storage for processing raw data and used by the mobile back-end via its controller. Like mobile app storage, it needs only a basic access control with username and password.
  - Database servers: These are the main storage, and contain all the BigO data for analysis. The data is available for a variety of end-users depending on their roles. A Role-based Access Control (RAC) mechanism is employed with specific policies for granting roles and permissions. Each database is accessed via a RESTful API. (Section 6.2 for details).

The BigO data privacy protections are based on the type of personal data (Table 2). These data types are given below:

- Inertial sensor, movement, and Mandometer data: These data types are collected and stored on personal devices (smartwatches, mobile phones, Mandometers). Only statistical and generalised data is extracted and submitted to the BigO server. These kinds of data do not raise privacy risks.
- Photographs: All the photos are reviewed by BigO admins. Any photo that is deemed to be irrelevant, indecent, or reveals the user’s identity is deleted from the system.
- Identifiable data: All attributes, such as username, deviceID, etc., are removed before storing the data in the data warehouse. The operation of removing such attributes is called de-identification (Section 6.3).
- Quasi-identifiable data: All data attributes, such as country, region, school, clinic, height, weight, gender, birth year, self-assessment answers, locations of photos, etc., are dealt with by anonymisation. However, because of the bad impacts on the data quality, a privacy-aware protocol is applied to take into account this type of data (Section 6.3).

### 6.1. Data Protection

Mobile app: The mobile app is built for both Android and iOS. Both operating systems have built-in security features. We exploit these features to protect the data collected by the app, even in the case where the default system and file permissions are used [17]. For instance, the core security features of Android OS include the Android Application Sandbox that isolates the application data and code execution from other applications. The same features were also offered in iOS.

The BigO application stores all data (including the SQLite DB data, the in-app acquired photographs, and the shared preferences) in the internal storage of the device. All that data is deleted as soon as it is synchronised/uploaded into the BigO server.

Database servers: MongoDB and Cassandra systems incorporate very interesting security models. For instance, the MongoDB security model is divided into four main parts: authentication, authorisation, auditing, and encryption. We explain briefly how data security protection features are supported by MongoDB and Cassandra in the following.

- Authentication: MongoDB integrates external security mechanisms including Lightweight Directory Access Protocol (LDAP) [40], Windows Active Directory, Kerberos [41], and x.509 PKI [42] certificates to re-enforce the access control to the database.
- Authorization: User-defined roles can be defined in MongoDB to configure granular permissions for a user or an application based on the privileges they need. Moreover, one can define views that expose only a subset of data from a given collection.
- Auditing: For regulatory compliance, MongoDB security model records native audit log to track access and operations performed against the database.
- Encryption: MongoDB security system offers data encryption data on the network, disk, and backups. By encrypting database files on disk, one eliminates both the management and performance overhead of external encryption mechanisms.
- Monitoring and Backup: MongoDB ships with a variety of tools, including Mongostat, Mongotop, and MongoDB Management Service (MMS) to monitor the database. Sudden peaks in the CPU and memory loads of the host system and high operations counters in the database can indicate a denial of service attack.

Apache Cassandra is a NoSQL database system and it is not based on a shared architecture, such as MongoDB. It relies on DataStax Enterprise (DSE) [43] for providing security features such as Encryption of data in-flight and at-rest, Authentication, Authorization, and Data auditing. DataStax Enterprise (DSE) [43] integrates cohesively with the existing technology estate, including support for Active Directory (AD), the Lightweight Directory Access Protocol (LDAP), Kerberos, Public Key Infrastructure (PKI), and Key Management Interoperability Protocol (KMIP). They are explained below.

- Encryption: It maintains data confidentiality. Usually, DB data encryption falls into two categories: Encryption At-Rest and Encryption In-Flight. The first refers to the protection of data that is stored on persistent storage. The second refers to the encryption of data as it moves over a network between nodes or clients and nodes within a DSE cluster.
- DSE Transparent Data Encryption (TDE): is the feature responsible for the encryption of at-rest data in a DSE system. DSE TDE protects sensitive at-rest data using a local encryption key file or a remotely stored and managed Key Management Interoperability Protocol (KMIP) encryption key.
- Authentication: refers to the process of establishing the identity of the person or system performing an operation against the database. DSE Unified Authentication facilitates connectivity to four primary mechanisms for authentication, as described below. It extends the same authentication schemes to the database, DSE Search, and DSE Analytics.
- Authorisation: In DSE, the authorizations determine which resources (i.e., tables, keyspaces, etc.) can be read, written, or modified by a connected entity, as well as their connection mechanisms. It uses the GRANT/REVOKE paradigm for authorization to prevent any improper access to the data and uses three mechanisms for user authorisations: Role-Based Access Control (RBAC), Row-Level Access Control (RLAC), Proxy Auth.
- Auditing: Data auditing allows to track and log all the user activities performed on the database to prevent unauthorised access to information and meet compliance requirements. With DSE, all or a subset of an activity that takes place on a DataStax cluster is recorded along with the identity of the user and the time the activity performed. Efficient auditing in DSE is implemented via the log4J mechanism that is built into the platform.
- Drivers: DataStax provides drivers for C/C++, C#, Java, Nodejs, ODBC, Python, PHP, and Ruby that work with any cluster size whether deployed on-premise or cloud data-centres. These drivers are configured with some features, such as SSL to ensure the users interact with the DSE clusters safely and securely.

#### 6.1.1. Auxiliary File Storage

The auxiliary file storage is used in BigO for storing raw accelerometer and gyroscope data for development purposes. Such inertial measurement data cannot be used to identify users and do not pose any privacy risk. The data is stored in the custom binary format in a secure Unix server.

#### 6.1.2. Data Transmission

Data is transmitted between (a) smartwatch and the mobile phone, (b) user mobile phones and the BigO servers, and (c) between different servers of BigO. The communication between the smartwatch and the mobile phone takes place over an encrypted Bluetooth channel and a potential attacker must be close to the user. Furthermore, the data is only accessible by the BigO app on the mobile phone; all other apps running on the phone cannot receive the transmitted data. Communication between the mobile phone and the BigO servers is encrypted using 2048-bit SSL. Finally, given that all the BigO servers are part of the same datacentre, the transmission between the servers is not critical.

### 6.2. Data Access Control

#### 6.2.1. Registration

The registration procedure depends on the user type. The different procedures are:

- BigO Administrator: This is created by the BigO developers and it is fixed. A BigO administrator can register the school and clinic administrators. The same can also review submitted pictures to remove inappropriate pictures or pictures that compromise the privacy of individuals.
- School Administrator can add/edit school details and register the teachers.
- Clinic Administrator can add/edit clinic details and register clinicians.
- Teachers can create groups, edit student groups and individual student details, such as BMI, school exercise schedule, etc., and can create registration codes for students.
- Clinicians can create registration codes for patients and edit individual patient details, such as BMI.
- Students can register through the BigO mobile app using a registration code provided by their teacher. When a teacher creates a student account, a registration code is generated and stored in the database. The student enters the registration code the first time s/he uses the app. Once the registration code is “redeemed”, the student is registered in the system and the registration code is no longer valid.
- Patients register with a registration code provided by their clinician; same as the students.

#### 6.2.2. Authentication:

User authentication takes place on a dedicated authentication server via JSON Web Tokens. The process is as follows:

- Each user has a username and a password (for students and patients, these are auto-generated and stored on the mobile phone, without the involvement of the user). The password is salted and hashed, and the encoded password is stored in the database.
- When the mobile phone needs to access a restricted REST endpoint, it first asks for JSON Web Token (JWT) from the authentication server, by presenting the user credentials. The credentials are also salted (with the same salt) and hashed, and the authentication server compares the encoded passwords. If they match, it provides the user with a valid JWT.
- Using the JWT, the mobile app and web portals/applications can access the restricted REST endpoints, until it expires. After expiration, the mobile app asks for a new JWT from the authentication server and the process is repeated.

#### 6.2.3. Authorization:

The BigO roles include BigO administrators, school administrators, clinic administrators, teachers, clinicians, students and patients, public health authority role, and volunteer students. The access control is implemented at the application layer by the BigO controllers (Mobile-app controller, portals controller, web application controller). We also include role-based collection-level access control at the database layer (natively supported in MongoDB), as extra security and data protection mechanism.

### 6.3. Data Privacy Protection

In BigO, data are stored in the mobile app storage (including the mobile file system and the SQLite database), the auxiliary file storage, the Cassandra database, and the MongoDB database. Except for the data in the MongoDB database, the data in the other places is accessed and used by the data owners and the internal modules. The MongoDB database is rarely directly shared for analysis. This is to prevent shared data from disclosing sensitive information of data owners. Therefore, this section focuses on the privacy preservation of the MongoDB database when this data is shared for analysis purposes.

We first discuss the challenges of privacy protection in the next sections. Then we further discuss how the effects of these challenges are mitigated with the privacy-aware protocol.

#### 6.3.1. Deidentification and Pseudonymisation

Deidentification conceals the real identities of data owners by removing all fields that can directly identify an individual, such as name, phone number, and email. In our case, deidentification does not affect the quality of data analysis results.

When the data are de-identified and shared, a new random identifier is used to name individuals. In some cases, it is required to keep the relation between the old and new identifiers in order to update the de-identified data. Therefore, pseudonymisation is used to create pseudo-IDs for individuals and encrypts the linkage between the real and pseudo IDs. However, in the BigO system, “username” and “display_id” are used in the interfaces with end-users whereas children IDs are used only by the internal modules. Therefore, we do not need to create pseudo IDs for children when their data is shared. The children’s IDs can be kept to differentiate individuals in the shared dataset without disclosing their identity.

In addition, the fields which are not necessary for analysis can be removed at the same time. It is important to clarify that the database collection “Photos” is not considered as an identifiable information source. This is because uploaded photos are checked by face-recognition algorithms and the BigO admins to guarantee that they only contain food advertisements and meals.

#### 6.3.2. Anonymisation

Practically deidentification and pseudonymization are not enough to keep a dataset safe because of quasi-identifiers. Unlike identifiable fields, the removal of quasi-identifiers can impact the quality of data analysis. The privacy models, such as k-anonymity, l-diversity, and LKC-privacy for preventing record and attribute linkage attacks, use various anonymisation operations, such as generalisation, suppression, shuffling, perturbation, and adding noises. However, some operations (e.g., shuffling, cell generalisation, and perturbation) are only suitable for specific analysis and can make datasets unavailable for the mining algorithms.

BigO is a big data system with high-dimensional datasets, especially when multiple database collections are combined. Therefore, anonymisation, using k-anonymity and its expanded versions, on high-dimensional datasets causes serious data distortion leading to poor quality of data analysis.

#### 6.3.3. Privacy-Aware Data Analysis Protocol

**Figure 7:**
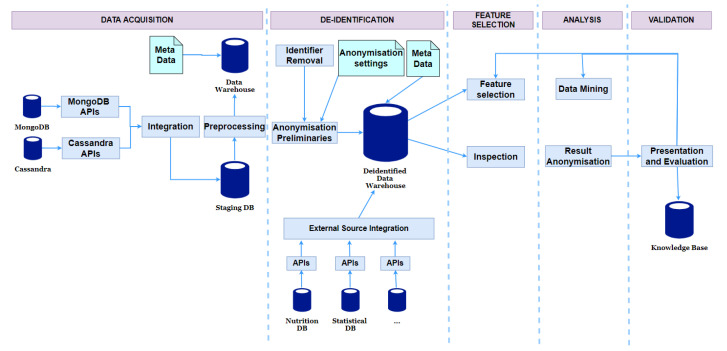
Privacy-aware data analysis protocol.

The proposed privacy-aware protocol is shown in Figure 7. The protocol is designed to deal with the problems of identifiable and quasi-identifiable attributes when sharing data for analysis and it considers the high-dimensionality issue.

In traditional approaches, the analysis tasks are applied to the anonymised data whose quality is degraded. The overall idea of the protocol is to de-identify but not anonymise the data. Instead of anonymising the data and sharing it for analysis, we provide special secured views for data scientists to inspect datasets and then run the analysis on de-identified but non-anonymised datasets. The special views enable these end-users to examine the data from various perspectives but not reveal the linkage between sensitive information and patients. The data scientists can choose feature selection methods and analysis algorithms that are run on the de-identified but non-anonymised data. Since the data does not contain identifiers, the discovered results are of high quality. Furthermore, this running process is managed by the system and the scientists cannot access the non-anonymised data. Before being released, the results are checked for non disclosing unexpected information. Some details of the resulted models are filtered to guarantee privacy for children. The steps of the protocol are as follows:

- Deidentification: The identifiable attributes and non-important attributes for analysis are removed in this step.
- Anonymisation Preparation: Some attributes require minor treatments that support generating secured views. This case often occurs to date and numerical but not categorical attributes. For example, it is usually not essential to keep detailed height values. So we round them off to ranges. This transformation is not an anonymisation operation and it just slightly changes the data content and the information in the data for analysis is nearly preserved. Hence, we consider this step as anonymisation preparation. Another important job of this task is to create anonymisation preliminaries (including taxonomy trees for quasi-identifiable attributes) which are used to generate secured views.
- Secured Views Generation: Secured views are created to help data scientists inspect and understand the dataset from a variety of perspectives but not reveal the linkages between the patients and their sensitive information. There are three types of secured views:
  - Statistical View: This provides measures, such as standard deviations, domain ranges, and value statistics for attributes being calculated automatically.
  - Anonymised View: This provides the whole view of shared datasets. For privacy protection, we applied the Privacy and Anonymity in Information Security (PAIS) algorithm [44] to achieve the LKC-privacy model [45]. LKC-privacy prevents record and attribute linkage attacks for high-dimensional datasets. PAIS uses the top-down searching strategy on taxonomy trees to find sub-optimal generalisation for records. For general analysis tasks, discernibility cost is used as the measure to choose the best specialisation.
  - Anatomised View: Since k-anonymity is a condition of LKC-privacy, the results of PAIS suffer from the problem of high-dimensionality. As a consequence, anonymised views may provide too general views on quasi-identifiers. Detailed or anatomised views are also provided using the anatomy technique.
- Feature Selection: After examining the datasets with different views, the data scientists can choose appropriate transformation, feature selection, and extraction methods to generate proper input data for their application-specific analysis tasks. The processing is done on the de-identified and non-anonymised dataset.
- Data Mining and Result Anonymisation: Data scientists can choose various analysis methods. The returned results can be too detailed in some cases. For instance, a decision tree (the output of the above classification algorithm) has detailed leaf nodes that link to several special individuals. Therefore, the mined results must be checked and filtered before being released for researchers to guarantee children’s privacy.
- Presentation and Evaluation: The resulting models are evaluated, and the data analysts can be restarted their analysis from the inspection step if necessary.

## 7. Implementation for Privacy-Aware BigO System Architecture

### 7.1. Description of Architectural Changes

**Figure 8:**
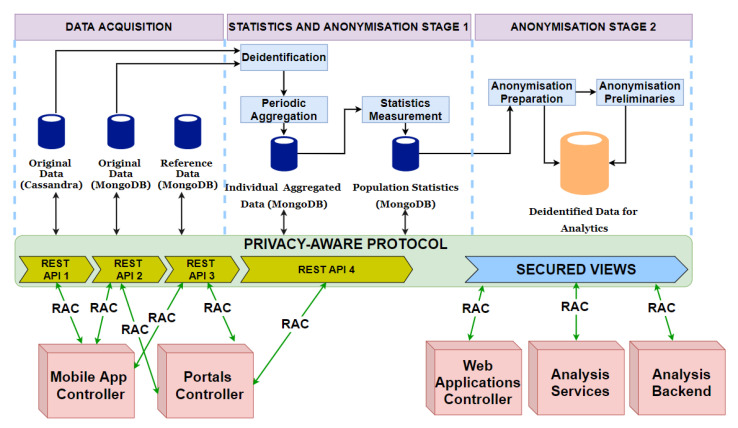
Privacy-aware BigO architecture.

To employ the privacy-aware protocol, described in Figure 7, into BigO architecture (Figure 6), we updated the architecture as shown in Figure 8. It can be seen that some intermediate modules are added between de-identification and anonymisation preparation steps. These modules support the periodic aggregations and population statistics pre-computation. The main updates of the architecture are listed below:

- Separation of the MongoDB database: Unlike the BigO component diagram (Figure 6) with one MongoDB database, the collections of this database are separated into three databases:
  - First MongoDB database (Original data): This database contains administrative data and collected/measured data, including collections USERS, CHILDREN, MEALS, TIMELINES, FOOD_ADVERTISEMENTS, DAILY_ANSWERS, and PHOTOS.
  - Second MongoDB database (Reference data): This database stores data unrelated to individuals and used for reference. The list consists of collections SCHOOLS, CLINICS, GROUPS, REGIONS, and PUBLIC_POIS.
  - Third MongoDB database (including Individual Aggregated Data and Population Statistics): Individual Aggregated Data includes collections summarising periodically behavioural data of individual children such as DAILY, WEEKLY, and STATISTICS. Population Statistics Data comprises collections COUNTERS, PUBLIC_POIS_VOTES, GEOHASH_VOTES, GEOHASH_ATTRIBUTES, and HISTOGRAMS.
- Separation of APIs: The updated BigO architecture supports four different APIs to access the Cassandra database and the three MongoDB databases.
- De-identification module: This module removes identifiable fields as well as the fields unnecessary for analysis and does not require pseudonymisation.
- Periodic aggregation module: This module aggregates periodically the behavioural data of children.
- Statistics measurement module: This module pre-computes statistics of some populations that are used for visualisation features and generating the statistical view.
- Anonymisation preparation module: This module conducts the tasks described in the Anonymisation Preparation step of the aforementioned privacy-aware protocol. The outputs of this module are Anonymisation Preliminaries and de-identified data for analysis.
- Anonymisation preliminaries: These are saved in the format of JSON or XML.
- De-identified data for analysis: This data storage should not store discrete collections like in the database of Individual Aggregated Data and Population Statistics. The data for analysis should be the combined datasets in the formats convenient for generating secured views and mining algorithms. A good choice is CSV files stored in the file system storage of Hadoop.
- Secured views: The data for analysis is accessed through secured views. There are modules taking responsibility for generating secured views and running feature selections/mining algorithms on the de-identified data for analysis.

### 7.2. Process of Updating Data Changes

It is important to check when there are changes in the original data storage. In practice, there are two kinds of possible data updates:

- Changes of administration data: The administration data (e.g., email, name, address) is entered manually so that sometimes there are mistakes requiring updates. Since this data type is not extracted to be stored in other databases, the synchronisation is not a problem.
- Insertion of new measures: The behavioural measures are uploaded from the mobile app to the original databases frequently. After certain periods of time, to reflect the changes in the original data, the new summarised data is added into the individual aggregated data storage and the existing statistics are updated in the Population Statistics storage. The anonymisation preliminaries and de-identified Data for analysis are also re-computed.

## 8. Current Picture, Recommendations, and Future Directions

In this section, we review the lessons learned and our recommendations for implementing privacy-aware big data warehouse architecture and the steps towards further research directions.

We first highlight some of the major problems in the existing data warehouse architectures, including the lack of ability to deal with the curse of high-dimensionality. In traditional frameworks, the analysis tasks are applied to anonymised data where the quality of data is degraded. Our work presents the BigO data warehouse architecture and further integrates a novel privacy-aware protocol to deal with the problems of identifiable and quasi-identifiable data attributes while sharing the data for analysis tasks. We present the security protocols for data acquisition, integration, and evaluation at the two different levels, that is, the application level and the database level. We also present the data access control protocols for phases, such as registration, authentication, and authorization.

The key idea is to de-identify personal data but should not be anonymised. BigO data warehouse architecture uses secured views for the data scientists to mine the datasets. The special views enable the end-users to carry our exploratory tasks without revealing the linkage between sensitive information and corresponding patients.

The privacy and security protocols implemented in the BigO system can be applied to any data-driven application apart from the healthcare sector, where there is a risk for sensitive personal information loss during the data acquisition, storage, transmission, and access while carrying out the analysis tasks. The essence of these protocols is that without degrading the quality of data, one should be able to perform mining and analysis tasks on data without a threat of sensitive information losses.

As future work, we plan to define appropriate and accurate data governance policies and procedures to avoid any data breaches, violation of any individual’s privacy, and produce high-quality results for the benefit of the society and its citizen. Data governance is important as the BigO project deals with sensitive personal and children data that need to be managed and manipulated with a high level of care and trust. Therefore, the data governance life cycle methodology must ensure confidentiality, quality, and integrity of the data.

## 9. Conclusions

Since obesity has become a serious global public health problem with implications both on the individual and society at large, behavioural interventions and the environmental community factors must be taken into account to intervene in childhood. BigO uses citizen-scientist data collection methods and different technologies (smartphone, wristband, Mandometer) to create comprehensive models of obesity prevalence. Data acquisition in BigO allows researchers to create models that analyse behavioural risk factors and predict obesity prevalence, through associations with community behavioural patterns and local obesity prevalence.

Monitoring and storing of personal data makes the data representation, security, and access control is challenging task. This paper first implemented data access and storage components of the BigO including the interfaces with other system components to allow smooth functioning data aggregation, data analysis, and visualisation. We presented a three-layered flexible data warehouse architecture for BigO including a back-end layer, an access control layer, and a controller layer.

We further implemented the data representation and sharing protocols in the BigO databases and the storage models considering the privacy and security aspects. The data privacy and security plans are devised based on the types of collected personal data in the aspects of data storage, data transmission, and data access. We presented the challenges in privacy protection and implemented novel privacy-aware data analysis protocols to ensure that resultant models guarantee privacy for children. Finally, we implemented the BigO system architecture that integrates the aforementioned privacy-aware protocols.

## Figures and Tables

**Table 1:** Types of information in Electronic Health Records (I: identifiable, Q-I: quasi-identifiable, S: sensitive).

Attribute Types	Description	Examples	Privacy
Identifiers	Person identification	name, email, address	phone number *I*
Demographics	Person classification to a specific group of the population	race, age, gender, area, postal code, education, occupation, marital status	Q-I
Personal Biometrics	Medical information related to physical health	X-Ray, MRI, ultrasound, blood pressure, cholesterol, heart rate, allergies, ICU incidents, tests reports	*S*
Clinical information	Medical history	diagnoses, dosages, treatment services, medication, encounters, problems, therapies	Q-I, S
Mental information	Related to psychological, psychiatric, and psychosocial issues	sleep problems, psychology, excessive dieting, psychological sexual disorders	*S*
Life-style and activity information	Relevant to physical activities, life-style	physical activities, exercise regime, nutrition, energy consumption through exercises	*S*
Insurance and financial matters	Related to billing, reimbursements, insurance	DRG, financial class, primary and specialist providers	Q-I, S

**Table 2:** Data collection modes that deployed in the BigO system. All modes include the use of a smartphone. Abbreviations: Smartphone (SP), Wristband (WB), and Mandometer (MM).

Collected Data Type	Light	Standard	Enhanced
ine Accelerometry	SP	SP/WB	SP/WB
GPS	SP	WB (with GPS)	WB
Meal self-reporting	SP	SP	SP
Food Barcode scanning	SP	SP	SP
Food pictures	SP	SP	SP
Meal eating behaviour	-	Limited MM use	Extended MM use

**Figure A1:**
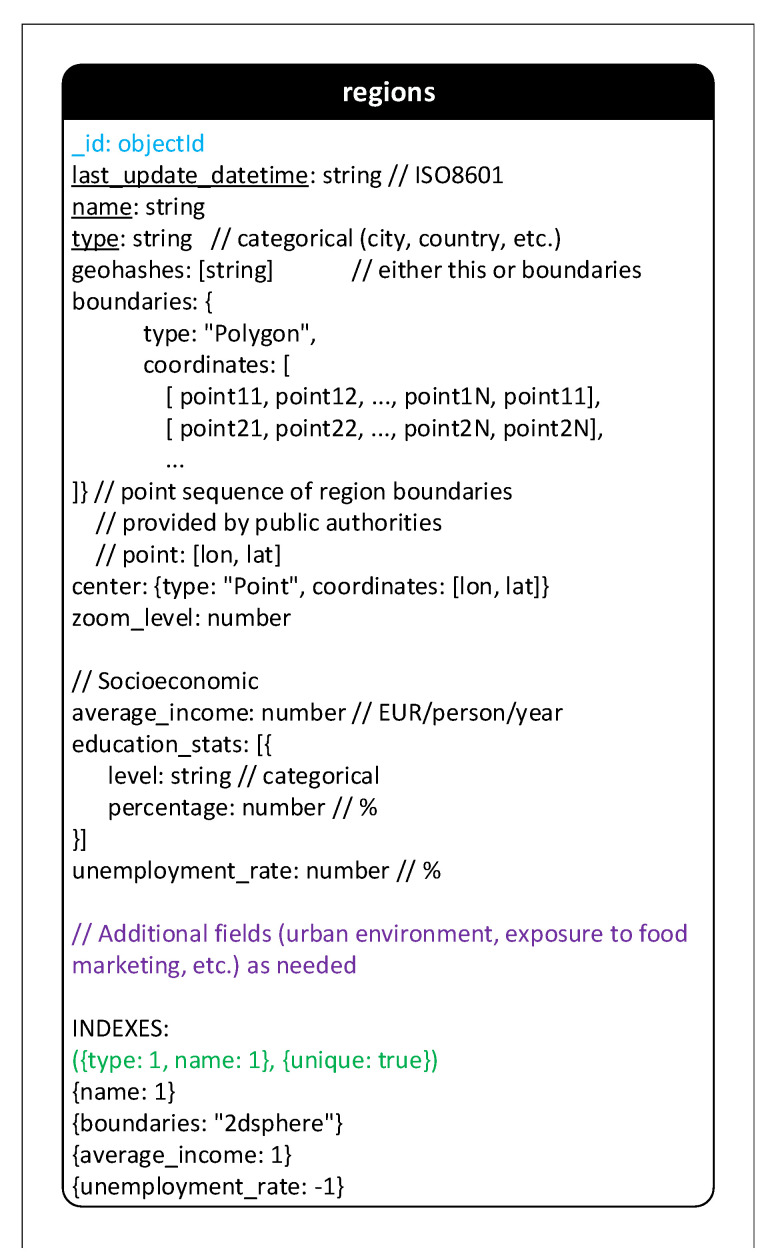
MongoDB Schema—Part 1.

**Figure A2:**
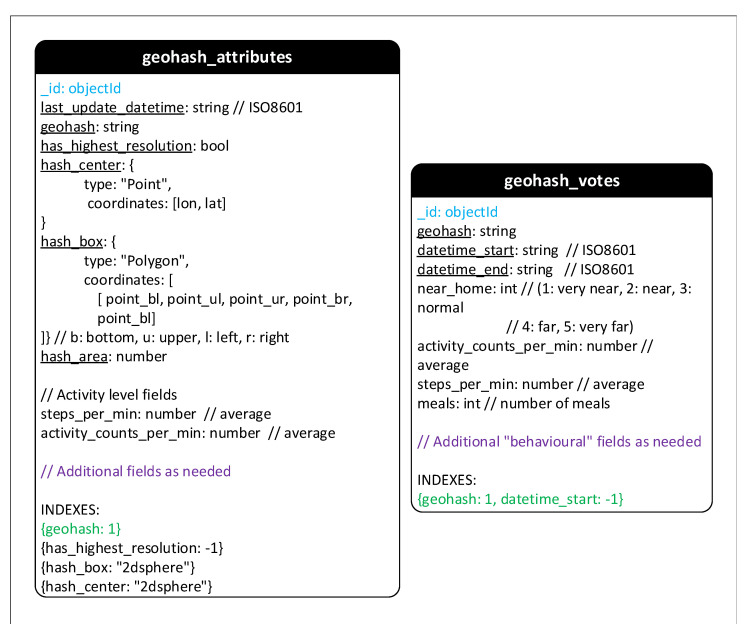
MongoDB Schema—Part 2.

**Figure A3:**
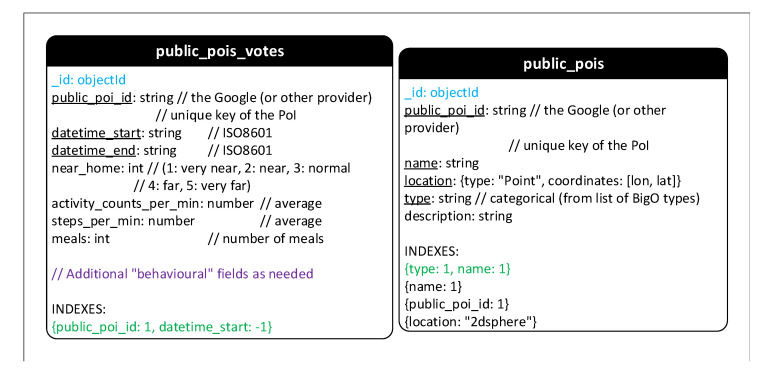
MongoDB Schema—Part 3.

**Figure A4:**
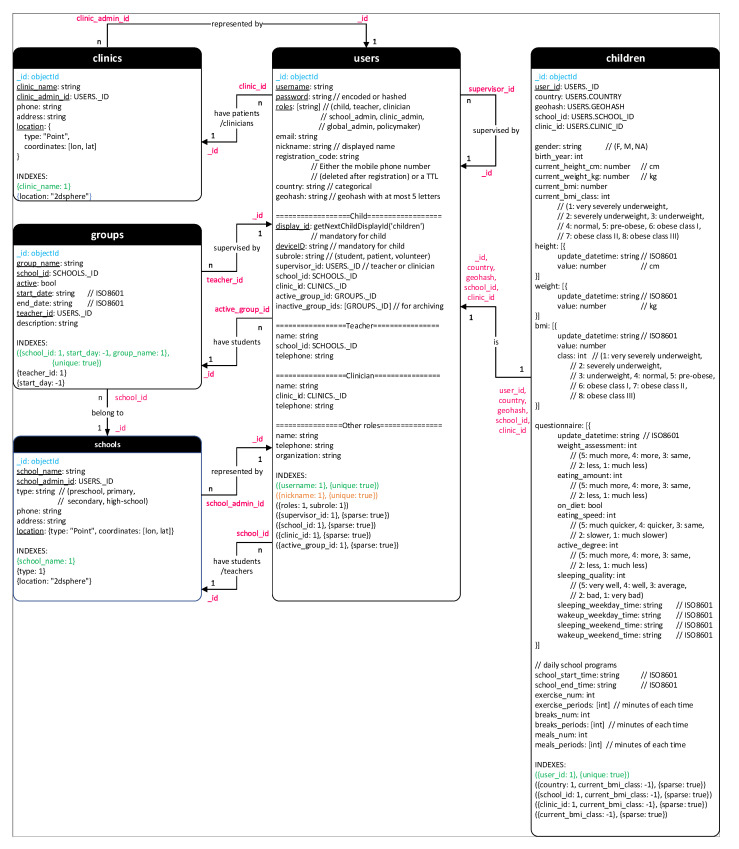
MongoDB Schema—Part 4.

**Figure A5:**
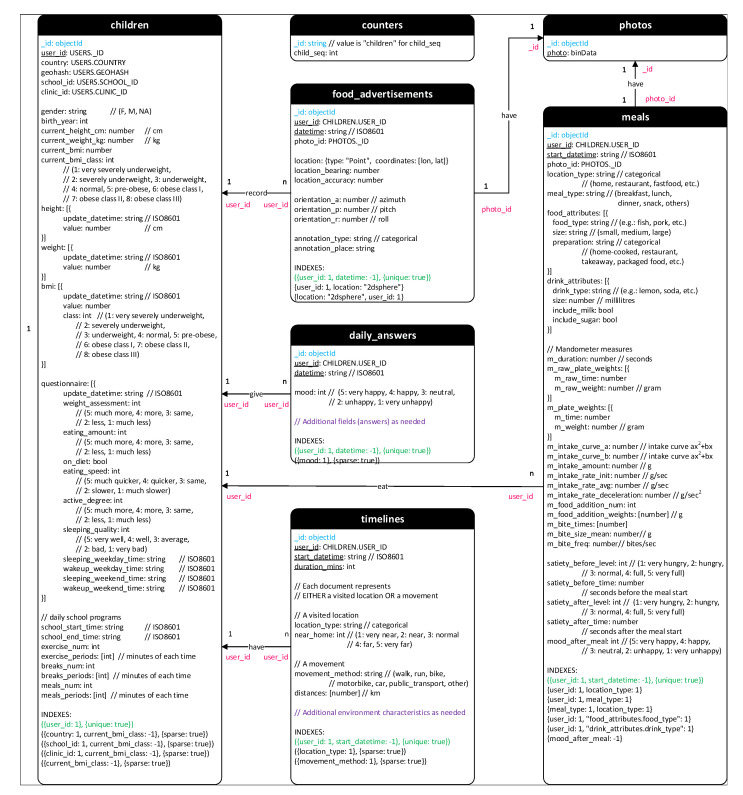
MongoDB Schema—Part 5.

**Figure A6:**
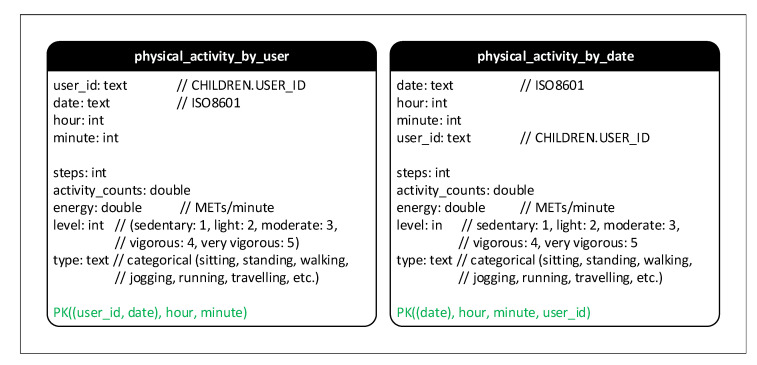
Cassandra database Schema.

## Data Availability

Data sharing is not applicable to this article.

## Abbreviations

The following abbreviations are used in this manuscript:

BigO	Big Data for Obesity
GPS	Global Positioning System
HIPAA	Health Insurance Portability and Accountability Act
EHR	Electronic Health Records
PHA	Public Health Authorities
LEC	Local Extrinsic Conditions
GIS	Geographic Information System
DBMS	Database Management System
API	Application Programming Interface
JWT	JSON Web Tokens

## Appendix A. Schema Design

This section present schema design for the core database in BigO.

### Appendix A.1. MongoDB Schema

Figure A1, Figure A2, Figure A3, Figure A4 and Figure A5 show the schema of MongoDB. The schema is organized in collections as follows:

- Regions: This collection stores interesting features of administration regions to represent all types of geographical or administrative areas. Therefore, a region can be a town, a city, a province or even a country. Each region has a series of coordinates that form its boundaries. The boundary information will be provided by public authorities. Depending on region type, fields of region characteristics can be added later.
- Geohash: Owing to the special characteristics such as the hierarchical structure and the ease of storing in databases, geohash is used in BigO system to represent geographical information. The data of children activities at some areas are recorded. However, only anonymous and statistic data are stored in collection Geohash_attributes and Geohash_votes.
- Public POIs: Two collections Public_pois and Public_pois_votes store aggregated activity information of children at public points of interest. Hence, the structure of collection Geohash_votes and Public_pois_votes are nearly the same.
- Schools and Clinics: These collections store the information of the organized schools and clinics.
- Groups: Students who take part in BigO system via the organized schools are divided into groups. Each group is managed by a teacher.
- Users and Children: BigO system has many types of users but only children data is collected for research. Therefore, Users collection is used to manage administrative fields while Children collection includes fields recorded for research. The available fields of a user depend on his/her role. Some special fields are shared since they are necessary for popular queries over the two collections. This duplication requires a small extra cost when inserting a new child but can improve the performance for many operations.
- Photos: Fields “photo” in collections Meals and Food_Advertisements in the previous version are separated to store in collection Photos. Grouping all photos in a specific collection smooths the management and verification of photos.
- Timelines and Mobility: Collection Mobility in the old version is replaced and extended to collection Timelines. The new collection contains not only the visited location but also the traveling activities of children.
- Daily_anwsers: This new collection contains the answers of daily questions.
- Counters: It is required to generate display IDs for children (in collection Users). These IDs are generated from an auto-incrementing sequence calling function getNextChildDisplayId(“children”). Once being called, this simple function adds 1 to field “child_seq” of the document having “_id” = “children” in collection Counters and return the new value as the new display ID. The similar technique and collection Counters can be used to create other auto-incrementing sequence.
- Sleeps, Mobility, Meals, and Food advertisements: These collections store data of daily activities of children. Especially, meals and food advertisements included photos taken by children.
- Eating habits: This collection contains the information of eating habits that can be extracted and aggregated from Meals.
- Statistics: The collection is used to store statistical information, such as the number of photos.
- Daily and Weekly: The data of activities can be aggregated daily and weekly and then stored in these collections.

### Appendix A.2. Cassandra Schema

Figure A6 shows collections in Cassandra. We now list them below:

- Physical_activity_by_user: stores the data of activities (i.e., walking, standing, sitting, running, etc.) for each user.
- Physical_activity_by_date: stores the data of activities (i.e., walking, standing, sitting, running, etc.) of users for each specific date and time.
